# Increased Visual Sensitivity and Occipital Activity in Patients With Hemianopia Following Vision Rehabilitation

**DOI:** 10.1523/JNEUROSCI.2790-20.2021

**Published:** 2021-07-14

**Authors:** Sara Ajina, Kristin Jünemann, Arash Sahraie, Holly Bridge

**Affiliations:** ^1^Department of Neurorehabilitation and Therapy Services, National Hospital for Neurology and Neurosurgery, Queen Square, London WC1N 3BG, United Kingdom; ^2^Wellcome Centre for Integrative Neuroimaging, Functional MRI of the Brain, Nuffield Department of Clinical Neurosciences, University of Oxford, Oxford OX3 9DU, United Kingdom; ^3^Division of Clinical Psychology and Sexual Medicine, Department of Psychiatry, Social Psychiatry and Psychotherapy, Hannover Medical School, 30635 Hannover, Germany; ^4^School of Psychology, University of Aberdeen, Kings College, Old Aberdeen AB24 3FX, United Kingdom

**Keywords:** cortical blindness, functional MRI, hemianopia, perimetry, rehabilitation, V5/hMT

## Abstract

Hemianopia, loss of vision in half of the visual field, results from damage to the visual pathway posterior to the optic chiasm. Despite negative effects on quality of life, few rehabilitation options are currently available. Recently, several long-term training programs have been developed that show visual improvement within the blind field, although little is known of the underlying neural changes. Here, we have investigated functional and structural changes in the brain associated with visual rehabilitation. Seven human participants with occipital lobe damage enrolled in a visual training program to distinguish which of two intervals contained a drifting Gabor patch presented within the blind field. Participants performed ∼25 min of training each day for 3–6 months and undertook psychophysical tests and a magnetic resonance imaging scan before and after training. A control group undertook psychophysical tests before and after an equivalent period without training. Participants who were not at ceiling on baseline tests showed on average 9.6% improvement in Gabor detection, 8.3% in detection of moving dots, and 9.9% improvement in direction discrimination after training. Importantly, psychophysical improvement only correlated with improvement in Humphrey perimetry in the trained region of the visual field. Whole-brain analysis showed an increased neural response to moving stimuli in the blind visual field in motion area V5/hMT. Using a region-of-interest approach, training had a significant effect on the blood oxygenation level-dependent signal compared with baseline. Moreover, baseline V5/hMT activity was correlated to the amount of improvement in visual sensitivity using psychophysical and perimetry tests. This study, identifying a critical role for V5/hMT in boosting visual function, may allow us to determine which patients may benefit most from training and design adjunct interventions to increase training effects.

**SIGNIFICANCE STATEMENT** Homonymous visual field loss is a common consequence of brain injury and is estimated to affect more than 230,000 people in the United Kingdom. Despite its high prevalence and well-described impact on quality of life, treatments to improve visual sensitivity remain experimental, and deficits are considered permanent after 6 months. Our study shows that behavioral changes following vision rehabilitation are associated with enhanced visually-evoked occipital activity to stimuli in the blind visual field. Unlike previous behavioral studies, we observe clinical changes that are specific to the trained region of vision. This lends significant weight to such training paradigms and offers a mechanism by which visual function can be improved despite damage to the primary visual pathway.

## Introduction

Hemianopia is a loss of vision on one side of visual space in both eyes, following postchiasmatic lesion along the visual pathway. In the majority of cases, it is caused by stroke in the territory of the middle or posterior cerebral arteries, although trauma or elective surgery in occipital cortex can also contribute to vision loss. The abrupt loss of vision can lead to reduced independence, inability to drive, difficulties navigateing in crowded environments, and potentially a loss of economic productivity. Despite the potentially significant effects of visual field loss on patients' activities of daily living, there is a lack of systematic access to visual rehabilitation through primary health services. A limited number of therapies are aimed at improving eye movement efficiency based on visual exploration (Sahraie et al., 2020; Szalados et al., 2020) or multisensory audiovisual training (Bolognini et al., 2005; Keller and Lefin-Rank, 2010). Although effective in improving patients' interaction with their environment, none of these therapies change the sensitivity of the visual system within the field damage (Rowe et al., 2017).

Initial attempts to improve sensitivity within the visual field deficit (restorative approaches) proved controversial, although more recently there have been encouraging results using a variety of stimulus training types. Some have involved repeated stimulation of visual deficits extending beyond the blind/sighted boarder and deep into the visual deficit (Huxlin et al., 2009; Sahraie et al., 2013). These techniques have led to improvement measured both in psychophysical testing (Sahraie et al., 2006; Raninen et al., 2007; Chokron et al., 2008; Huxlin et al., 2009; Sahraie et al., 2010) and more recently in visual fields (Elshout et al., 2016; Bergsma et al., 2017; Cavanaugh and Huxlin, 2017), although there is skepticism that the results may reflect practice effects and do not translate to a meaningful improvement in visual function. To put the results in context, patients with incomplete hemianopia might expect a mean deviation −15 ± 2 (dBs) on Humphrey perimetry, whereas the intact field scores above zero when compared with healthy age-matched controls (Sansal Gedik, 2007). Changes after visual training tend to be very small, with gains of 1 ± 0.3 dB (Elshout et al., 2016; Cavanaugh and Huxlin, 2017), if at all (Sahraie et al., 2013). However, this reflects an average across the entire visual field, and even small changes to mean deviation in glaucoma or idiopathic intracranial hypertension have been classified as meaningful (Chauhan, 2008). Improvements on perimetry also tend to be greatest in areas of retained visual sensitivity >0 dB (Elshout et al., 2016). Commonly, this involves the scotoma border zone, even if outside the region targeted by training (Cavanaugh and Huxlin, 2017). Improvements in trained regions that are deeper into the scotoma tend to be considerably weaker on perimetry (Huxlin et al., 2009; Sahraie et al., 2013; Elshout et al., 2016; Cavanaugh and Huxlin, 2017). This disparity remains unclear but may reflect a different mechanism of recovery in the border zone compared with deeper, denser areas of field loss.

Little is known about the neural changes that might underlie improvement in visual function with rehabilitation, compounded by the different patterns of recovery on perimetry versus psychophysical testing. It is possible that training improves activation in regions of early visual cortex that are adjacent to the damage, the perilesional cortex. Alternatively, the extensive training may improve residual visual function in areas outside V1, such as motion area hMT+, which has a role in blindsight (Cowey, 2004; Ajina et al., 2015c; Ajina and Bridge, 2018). A recent study found that extensive training on a motion discrimination task, which reduces visual field deficits, leads to an increase in the extent of visual neural activation in V1 (Barbot et al., 2020), although the recovery on this study was mostly confined to blind field border zones. This study also did not examine any extrastriate areas, so it is not known whether the training also induced changes elsewhere in the visual cortex.

In the current study, a group of volunteers with hemianopia were trained using the Neuro-Eye Therapy regime for 3–6 months. This training technique is associated with changes in visual sensitivity deep in the scotoma, at trained regions of the visual field (Sahraie et al., 2013). Psychophysics, visual field testing, and neuroimaging were performed both before and after training to quantify any changes in visual function and related neural underpinnings.

## Materials and Methods

### 

#### Participants

Seven participants with hemianopia (three female) caused by acquired brain injury took part in the training study. All had suffered a stroke at least 6 months before enrolling. Average age at the time of participation was 61.3 ± 13.1 years, and average time since lesion at first scan was 14 ± 7 months. An additional four participants with hemianopia (three female) took part in the control study. All had suffered a stroke at least 6 months before enrolling. Average age at the time of participation was 49.5 ± 14.3 years, and average time since lesion at first assessment was 17 ± 9 months. There was no difference in average age (*t* = 1.4; df = 9; *p* = 0.20) or time postlesion (*t* = 0.6; df = 9; *p* = 0.55) in the two groups. Written informed consent was obtained from all participants, and the research adhered to the Declaration of Helsinki. Ethical approval was provided by the Oxfordshire Research Ethics Committee (Reference B 08/H0605/156) or Oxford University Central Ethics Committee (Reference MS-IDREC-C2-2015–025).

#### Study design

Training participants participated in either two or three study sessions, each with the same format. A session included a magnetic resonance imaging (MRI) scan session, visual field testing, and visual psychophysics. After the first session, participants were provided with training apparatus in their home and undertook a visual training paradigm provided by Neuro-Eye Therapy. After a period of 4–5 months, participants returned to Oxford for the second scan session. Control participants participated in the same visual psychophysics testing before and after an equivalent period of 4–5 months but without training. This comparison was to rule out a practice effect of testing. However, functional MRI (fMRI) data were only acquired in the training group, not the control group, which means it is not possible to fully exclude a placebo effect on the MRI data.

#### Training protocol

**Figure 1.**
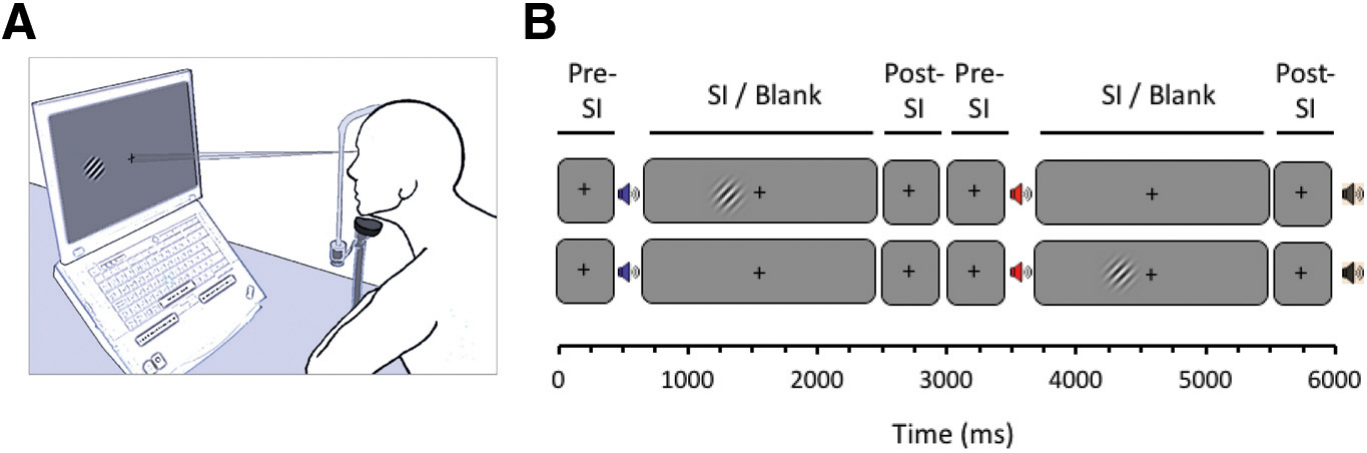
***A***, Rehabilitation setup in patients' homes. Participants sat with their head on a chin rest at a distance of 40 cm from the monitor, mounted on a frame. ***B***, The training task consisted of a 2-AFC temporal detection task in which a spatially and temporally modulated Gabor patch was presented in one of two intervals while the participant maintained central fixation. The target contrast was algorithmically controlled to maintain difficulty. Participants provided their response by pressing the left or right response buttons, and positive auditory feedback was provided if they were correct. Each training session lasted ∼25 min, with a 3 min rest imposed halfway through the session. SI, Stimulus interval.

The training procedure was conducted in the participant's home on an IBM compatible personal computer, mounted on a frame (Fig. 1). Gamma corrections were conducted on all monitors using an LS-100 Luminance Meter (Konica Minolta) at 256 equi-stepped logical colors. Participants sat with heads on a chin rest at a distance of 40 cm from the monitor, with line of sight approximately level with the fixation point. Viewing was binocular throughout the experiment.

**Figure 2.**
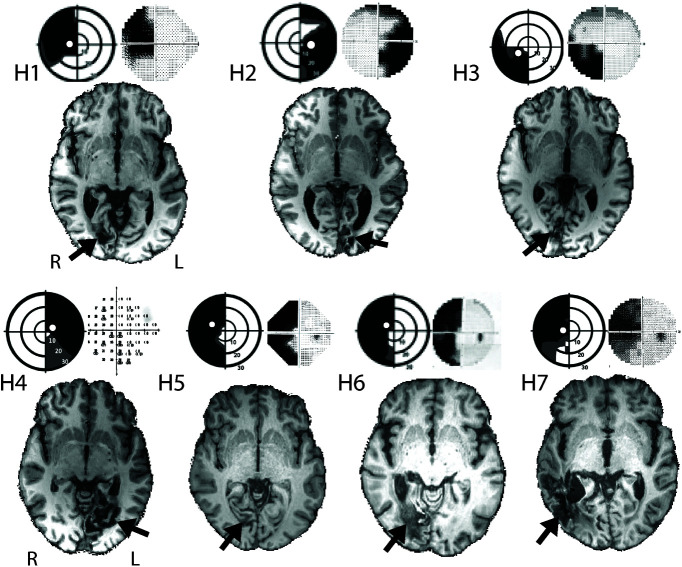
Structural images for all seven participants, with the location of damage indicated by the arrow. Visual field deficits are adapted from 30:2 threshold Humphrey visual field perimetry reports and show dense visual field loss in black (<0.5%) and partial loss in gray (<2%). Stimulus training and testing location (indicated by the white circle superimposed on the black region) was different for each participant and restricted to a region of dense visual field loss. Concentric rings represent increments in retinal position of 10°, spanning the central 30°.

Training stimuli consisted of achromatic Gabor patches of vertically oriented sine wave gratings, with spatial smoothing of the boundaries (spatial frequency = 1cyl/°; temporal frequency = 10 Hz; diameter = 6°). Stimuli were presented at three predetermined retinal eccentricities in a randomly interleaved order. The exact locations were tailored to each participant's deficit (Fig. 2; Table 1).One of the three locations overlapped with the test stimulus used in fMRI and psychophysical experiments, chosen to fit into the area visible from within the MRI scanner. This represented the primary target of the current rehabilitation study and was the location for all psychophysical assessments, performed before and after the training program. The targeting of three locations was standard protocol for the training apparatus to maximize the region of visual field undergoing rehabilitation.

**Table 1.** Participant training details

Participant ID	Time after lesion (months)	Training sessions	Assessment interval (days)	Training stimulus locations			Test location	Test diameter (degrees)
				A	B	C		
H1	19	37	217	−5,0	−10,0	−5,6	−6.5,0	5
H2	18	58	252	7,−3	10,3	4,−9	8,−3	8
H3	6	41	102	−12,−3	−6,−3	−18,−3	−12,−5	5
H4	7	59	393	6,4	12,4	6,10	6,4	5
H5	11	103	141	−11,6	−11,12	−11,18	−9,5	5
H6	26	48	127	−6,4	−6,10	−6,16	−6.3,4	5
H7	13	> 21	308	−8,0	−8,6	−8,−6	−7,0	8

Training and test stimulus locations are the *x* and *y* coordinates of the stimulus left in relation to the central fixation cross (0,0), in degrees.

The training task required detection of a Gabor patch using a temporal two-alternative forced-choice (2-AFC) task. Participants were required to report whether a target stimulus was presented during the first or second of two intervals. The intervals were separated by auditory cues of one or two announcing the start of each interval. Each trial lasted 6 s in total and finished with a low tone (Fig. 1). At the start of training the target contrast at all three locations were set to 95%. This contrast at each location was then lowered by 10% after three consecutive sessions when correct performance was above 84%. Reduction of performance to 64% and below resulted in an increase of contrast by 5% in the subsequent training session (Sahraie et al., 2010). This method has been shown to ensure maximum stimulation while increasing the task difficulty with improved performance. Auditory feedback was provided to denote correct discrimination.

#### Psychophysical testing

Two detection tasks using different psychophysical stimuli were used before and after the training period. Stimulus diameter was either 5° or 8° with the location restricted to the scotoma, and a minimum of 3° from fixation on a uniform gray background of luminance 50 cd/m^−2^. The tasks were as follows: (1) contrast: detect a drifting achromatic Gabor patch (temporal frequency 10 Hz, spatial frequency 1.3 cycles/°) of variable luminance contrast (1, 5, 10, 50, 100%) and (2) speed: detect moving black dots (luminance 0.5 cd/m^−2^) of variable speed (4°/s, 8°/s, 20°/s, and 32°/s).

A two-interval forced-choice paradigm was used, and participants were required to indicate whether the stimulus appeared in the first or second time interval. Onset of each interval was indicated by a 500 ms auditory tone, 300 Hz marking onset of the first interval and 1200 Hz for the second. Stimuli appeared for 500 ms with jittered onset of range 500–1500 ms while the participant fixated on a central black cross.

Two direction discrimination tasks were used in which participants were required to determine whether a single stimulus interval contained moving dots with horizontal or vertical motion. Stimuli appeared for 500 ms with jittered onset as above while the participant fixated on a central black cross. The tasks were as follows: (1) motion coherence: discriminate global direction of motion of a patch of moving black dots of variable motion coherence (0, 12.5, 25, 50, 75, 100%). Nonglobal motion direction was random. The patch contained dots at an average density of 8 dots/°^2^, moving at a speed of 5°/s. Each dot was 0.075° in diameter and had a limited lifetime of 12 frames, with frame rate 60 FPS. (2) Speed: discriminate direction of moving black dots of variable speed (4°/s, 8°/s, 20°/s, and 32°/s). Dots moved with 100% coherence and unlimited lifetime.

At the start of psychophysical testing before training, an identical static test stimulus was used to confirm that patients were unable to see the stimulus at its selected size and location in the visual field. This was done using a predicted aperture size and locus based on prior perimetry results. Stimulus location had to be restricted to the boundary of the fMRI display, which subtended 23° horizontally and 13° vertically. This influenced whether a 5° or 8° diameter stimulus was chosen, as the stimulus had to stay inside the blind field while remaining on screen. The stimulus of choice was an 8° diameter aperture, but if this was not possible, the stimulus was reduced to 5° diameter. If the criteria were unachievable using either stimulus size, the patient was excluded from fMRI study (patient H3). If the patient was able to see any part of the test stimulus while fixating on the central cross, the aperture was repositioned 0.5° deeper into the scotoma (according to the perimetry report) until the patient could no longer see any part of the stimulus. Fixation was recorded throughout psychophysical testing using an EyeLink 1000 eye tracker (SR Research), and any trials with eye position deviation >1 degree from fixation were excluded from analysis.

Repeated measures 2-way ANOVA tests were performed to quantify the effects of stimulus parameter (contrast, coherence, or speed) and training for each psychophysical test. Paired *t* tests were used for each experiment to quantify overall effect of training, comparing performance measured before and after training.

Participants were asked to provide anecdotal feedback on any changes they had experienced with training at the start of the post-training session of psychophysical testing. H2 reported thinking he had improved in his ability to see motion but was not sure if this had made a real difference in his everyday life. H3 reported making fewer spelling errors when using his iPhone. H5 felt that there had been an improvement to the central and top left portion of his vision and reported that he was picking up more things that he missed previously. H1 and H6 both thought that the training had been beneficial but found it hard to pinpoint any specific change. H7 reported that he could now count the lines on a speaker, which he could not do before training.

#### Magnetic resonance imaging

##### Functional MRI procedure

Stimuli were presented on a 1280 × 1040 resolution monitor at the back of the MRI scanner bore. Participants viewed the stimuli via a double mirror mounted on the head coil. The screen subtended a visual angle of 23° × 13°.

The experiment has been described in detail in Ajina et al. (2015b), but essentially the visual stimuli were drifting achromatic Gabor patches of 5° or 8° diameter displayed on a uniform gray background of luminance 50 cd/m^−2^, which was equal to the Gabor patch mean luminance. Spatial frequency was fixed at 1.3 cycles/°, and the patches drifted at 10 Hz. Five contrast levels were presented separately to each hemisphere, producing a 10-condition block design, with equivalent diameter and screen position to that used in behavioral testing (Fig. 2). In each block, a Gabor patch of the same luminance contrast was presented eight times, for a duration of 2 s with an interstimulus interval of 500 ms. The angle of drift was randomly allocated one of two orthogonal directions for each stimulus. A 10 s rest period followed each 20 s block. Each participant performed three runs, each lasting 300 s.

A central fixation cross was present throughout the scan sessions, and to maintain fixation, participants were required (during condition and rest blocks) to press a button every time the cross color changed from black to red. These changes occurred at random lasting 300 ms duration, and participants were instructed at the start to try not to miss any red crosses. An EyeLink 1000 eye tracker (SR Research) was used to confirm central fixation.

#### Data acquisition

Participants were scanned either on a 3T Siemens Verio scanner or a 3T Siemens Prisma, using a 32-channel head coil, with the same scanner used for both visits. A T1-weighted 1 mm^3^ isotropic resolution magnetization-prepared rapid acquisition gradient echo anatomical scan [echo time (TE), 4.68 ms; repetition time (TR), 2040 ms; field of view, 200 mm; flip angle, 8°] was acquired for each participant at each scan session.

Four hundred and fifty-six echo-planar imaging (EPI) functional volumes were acquired in a single fMRI scan; a scan duration of 15 min (T2**-weighted echo-planar-imaging, 34 sequential 3 mm slices; TR = 2000 ms; TE = 30 ms; field of view, 192 mm).

Preprocessing and statistical analyses were performed using tools from the Functional MRI of the Brain (FMRIB) software library (FSL; http://www.fmrib.ox.ac.uk/fsl). Nonbrain tissue was removed using the Brain Extraction Tool (BET; Smith, 2002), motion correction was performed using MCFLIRT (FMRIB's Linear Image Registration Tool with motion correction; Jenkinson et al., 2002), images were corrected for distortion of 5 mm, and high-pass temporal filtering (Gaussian-weighted least-squares straight-line fitting with σ = 13.0 s) was used. Functional images were registered to high-resolution structural scans using FLIRT (FMRIB's Linear Image Registration Tool; Jenkinson and Smith, 2001) and to a standard Montreal Neurologic Institute (MNI) brain template using FLIRT.

#### Functional MRI data analysis

For whole-brain analyses of blood oxygenation level-dependent (BOLD) activity, it was necessary to align participant brains to a uniform pathologic template, so the lesion was located in the right hemisphere, corresponding to a left-sided visual deficit. This required flipping both the structural and functional images of two participants (H2 and H4; Fig. 2) The 10 stimulus block types were entered as explanatory variables separately for the first two scan sessions, but the contrast of interest was the response across all contrasts compared with baseline. A higher-level mixed effects paired *t* test analysis was conducted using this contrast to compare the difference in activity before and after training across the group.

A region of interest (ROI) analysis was performed to investigate the percentage of BOLD signal change in motion area V5/hMT. Masks were generated in MNI space centered on the coordinates provided in Kolster et al., (2010) for middle temporal (MT) area, (−48, −75, 8) for left and (46, −76, 6) for right. A 7.5 mm sphere was generated around these coordinates to produce a mask of 2000 mm^3^ in each hemisphere, consistent with the volumes from Kolster et al. Masks were transformed to EPI space to calculate BOLD percentage changes.

#### Voxel-based morphometry

The structural data were analyzed with voxel-based morphometry (VBM) from the FSL library (Smith et al., 2004). First, structural images were brain-extracted using BET (Smith, 2002), and tissue-type segmentation was conducted using FMRIB's Automated Segmentation Tool-4 (Zhang et al., 2001). The resulting gray matter partial volume images and the respective mirror images were then aligned to MNI−152 standard space using FLIRT, followed by nonlinear registration using FMRIB's Nonlinear Image Registration Tool (Andersson et al., 2007). The resulting images were averaged to make a study-specific template, to which the native gray matter images were then nonlinearly reregistered. Registered partial volume images were then modulated (to correct for local expansion or contraction) by dividing by the Jacobian of the warp field. The modulated segmented images were then smoothed with an isotropic Gaussian kernel with a σ of 3 mm (∼8 mm full-width at half-maximum).

Permutation-based nonparametric inference within the framework of the general linear model was used to determine statistically significant differences between the two time points across individual participants (5000 permutations; Nichols and Holmes, 2002)).

#### Data availability

All anonymized data are available on request from the authors.

## Results

The occipital damage can be seen in the T1-weighted structural images shown in Figure 2. In each case the axial slice is through the calcarine sulcus. The resulting visual field loss is shown next to the image, along with the location where the training paradigm was run. There is significant variability in both the size of the lesion and the extent of visual field loss.

### Effects of training varies across participants

**Figure 3.**
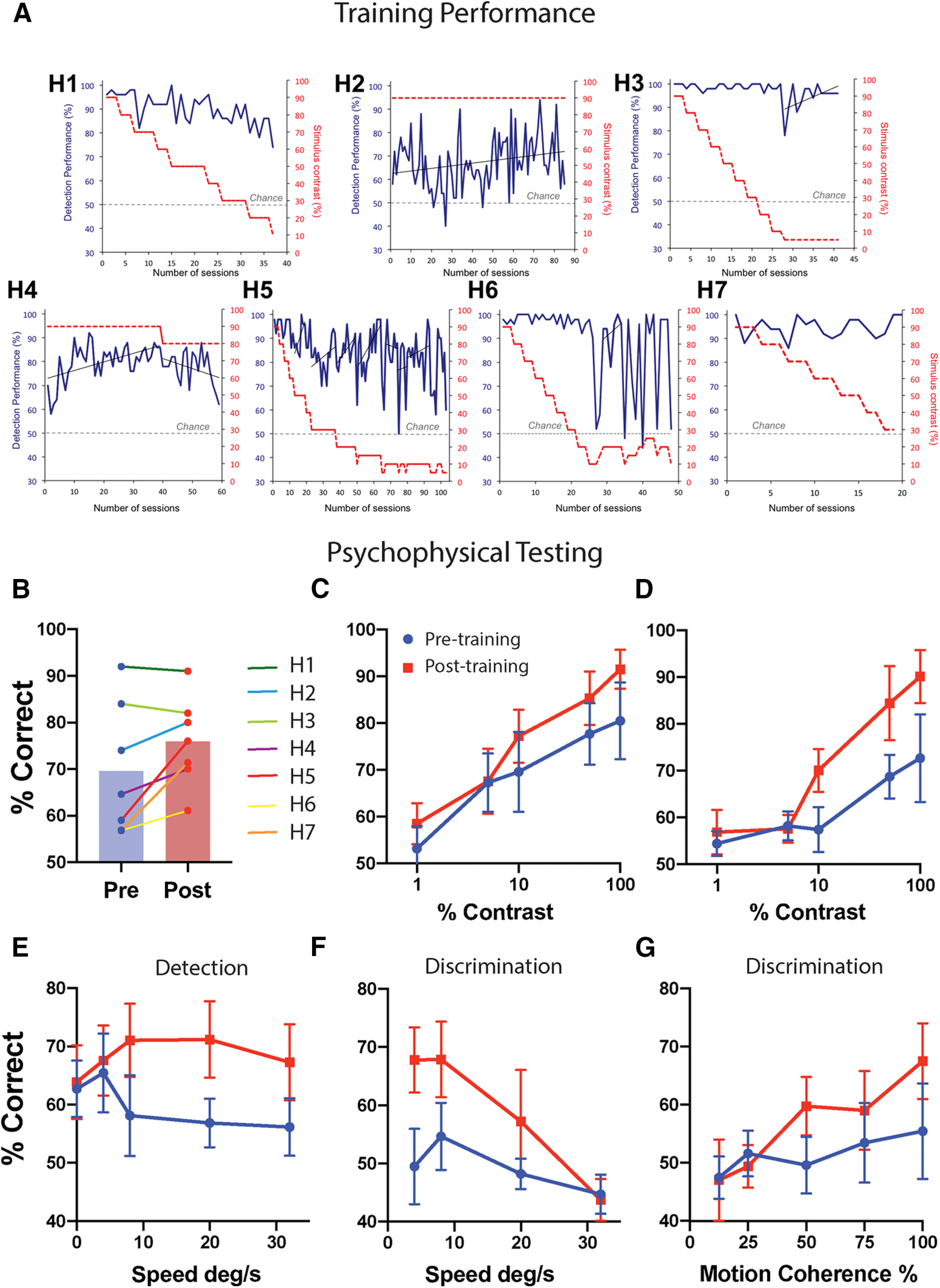
Training performance and behavioral improvement measured before and after training. ***A***, The adaptation of individual training parameters with performance over the home training period. Blue lines represent performance on the 2-AFC detection task throughout training (left *y*-axis), with the contrast of the training stimulus shown by the dotted red line (right y-axis). The stimulus contrast reduces when performance is consistently ≥84%, and ranges between 5 and 95%. Superimposed on the performance curves are lines of best fit (thin black lines), calculated for any period lasting >10 sessions duration where the contrast level remained constant. Where positive, this indicates an overall improvement in performance over time. Chance level is 50%, shown in gray. ***B***, The change in detection of a Gabor patch presented within the blind field averaged across all contrast values. The lines marked in green are participants who performed at ceiling on the high contrast stimuli before starting training. ***C***, ***D***, Performance on the contrast detection task at each contrast level; ***C*** includes all participants, whereas ***D*** excludes the two participants who performed at ceiling before training. E, Detection of moving dots presented within the blind field. ***F***, ***G***, Shows the ability to discriminate between horizontally and vertically moving dots. In each case, red data points represent behavioral performance after visual training, and blue is performance before the start of training.

Each participant completed a different number of sessions, depending on the period of training and the regularity with which they performed the sessions. Table 1 shows the number of sessions and duration of training for each individual. Figure 3*A* shows the training performance of each participant. As participants improved at the task, the stimulus contrast decreased to increase task difficulty. H1, H3, H5, and H6 all showed continuous improvement with training. H2 and H4 showed slight improvement. The high performance for H6 is at odds with the laboratory testing data for that participant. The training location appears to have included a small region at the boundary of the scotoma with weak but significant spared sensitivity (5 dB). Much of the training data from participant H7 were lost, and only a 3-week period at the beginning of training was available for inspection.

### All training participants show some improvement in visual performance

Performance was measured across the four psychophysical tasks. Figure 3*B* shows the overall performance on the detection of Gabor stimuli in the 2-AFC task (Table 2). Across all seven participants, there was a marginal effect of training (one-tailed Wilcoxen signed rank test; sum of ranks = 22; *n* pairs = 7; *p* = 0.04). The two highest performing patients at baseline performed equally well across pretraining and post-training (shown in light and dark green). The remaining patients all performed at significantly higher accuracy in the 2-AFC task at post-training compared with pretraining (one-tailed Wilcoxen signed rank test; sum of ranks = 15; *n* pairs = 5; *p* = 0.03).

**Table 2.** Summary of clinical, behavioral, and neuroimaging results in each participant

Participant ID	Lesion size (mm^3^)	Perimetry change at target (dB)	Contrast performance pretraining	Contrast performance post-training	% BOLD signal baseline	% BOLD signal change V5/hMT
H1	12735	3.04	92%	91%	0.05	0.16
H2	7830	0.14	74%	80%	−0.02	0.10
H3	2212	8.75	84%	82%	N/A	N/A
H4	12456	0.00	65%	70%	−0.11	0.09
H5	1472	4.04	59%	76%	0.08	0.17
H6	6299	−1.50	57%	61%	−0.25	0.27
H7	25137	5.06	57%	71%	0.31	−0.01

The effect of stimulus contrast on performance is shown in Figure 3*C*,*D*. Figure 3*C* includes all participants, and a repeated measures ANOVA showed a significant effect of stimulus contrast (*F*_(4,30)_ = 4.4; *p* = 0.006) and time point (*F*_(1,30)_ = 6.9; *p* = 0.013), indicating that performance was higher after training. There was no interaction between stimulus contrast and time point (*F*_(4,24)_ = 0.6; *p* = 0.7). Figure 3*D* excludes the two participants who performed at ceiling before training, as they would not be sensitive to improved performance.

Figure 3*E* shows performance on the detection of coherent moving dots at different speeds. There was no effect of stimulus speed on performance or interaction, but there was an improvement with training (*F*_(1,20)_ = 7.0; *p* = 0.015). For discrimination of direction of moving dots (Fig. 3*F*), speed of motion had a significant effect on performance (*F*_(3,39)_ = 4.6; *p* = 0.007). Additionally, there was significant improvement of direction discrimination with training (*F*_(1,27)_ = 7.6; *p* = 0.01), although the interaction between speed and time point was not significant. The proportion of dots moving coherently (Fig. 3*G*) showed a weak effect on motion direction discrimination (*F*_(4,36)_ = 2.4; *p* = 0.07. There was no effect of time point on discrimination of motion direction or interaction with stimulus coherence.

As a comparison, performance in the sighted field was also recorded. This was at ceiling (100%) in all detection tasks, pretraining or post-training, including stimuli with low luminance contrast. Performance was also at ceiling in speed discrimination, where participants had to determine whether dots of differing speeds were moving horizontally or vertically at 100% coherence. For the motion coherence discrimination task, performance was between 95 and 100% in the sighted hemifield at 50–100% coherence, pretraining and post-training. The lowest coherence stimulus (12.5%) elicited mean performance 76.7% ± 6.7% and 63.3% ± 8.8% pretraining and post-training, respectively.

The control participant group showed no improvement in performance after an equivalent time period across any of the four psychophysical experiments. This included 2-AFC detection of Gabor stimuli (76.5% ± 5.4% pretraining vs 69.25% ± 8.7% post-training), detection of moving black dots (59.0% ± 5.6% vs 47.1% ± 4.7%), discrimination of motion direction for moving dots controlled for speed (58.1% ± 4.4% vs 51.1% ± 3.6%), or for moving dots controlled for percentage coherence (43.3% ± 3.7% vs 48.7% ± 1.8%). This suggests that improvement in the training group was unlikely to be a result of spontaneous recovery nor a practice effect from repeated participation in psychophysical blindsight testing.

### Increased neural activity to motion stimuli after training

**Figure 4.**
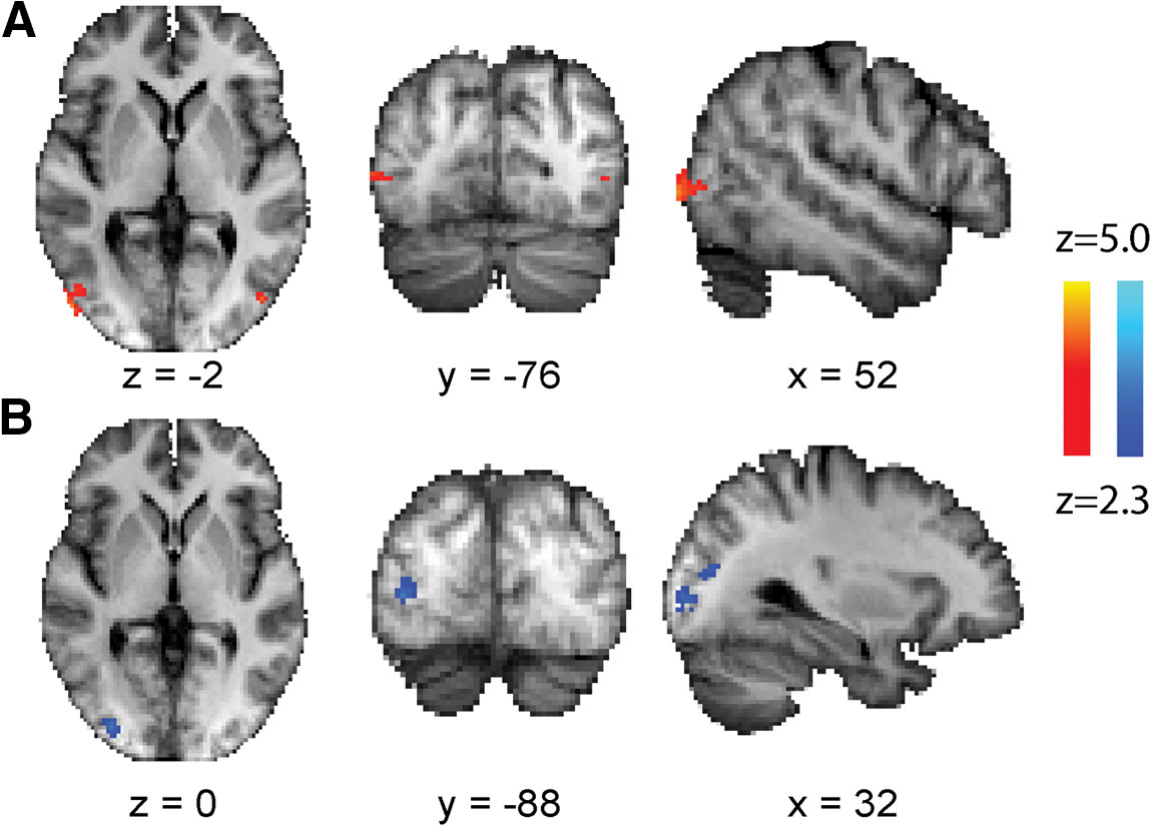
Increase in activity in area V5/hMT to the contrast stimulus averaged across all conditions in the blind field after training, compared with before training. ***A***, Although there is an increase in activity in the damaged hemisphere to stimuli presented in the blind hemifield, there is also a decrease in response in that hemisphere to stimuli presented in the sighted field, that is, a decrease in ipsilateral activity after training (***B***). Mixed effects analysis, *p* < 0.001 uncorrected for a priori regions of interest in the occipital lobe, elsewhere cluster corrected *p* < 0.01.

Six of the participants were scanned using fMRI before and after training. Participant H3 participated in the MRI scan session, but the location of visual field loss meant that it was not possible to place the stimulus fully within the scotoma, and fMRI data could not be acquired. Figure 4*A* indicates the region that showed an increase in activity between the pretraining and post-training scan sessions, averaged across all contrast levels. The data shown are the group analysis of post-training BOLD activity minus pretraining BOLD activity for each participant analyzed using a paired *t* test. The only region showing a significant increase in activity is V5/hMT in the trained, lesioned hemisphere when stimuli are presented to the blind field. Interestingly, when stimuli are presented to the sighted field, there is a decrease in activity in the trained, lesioned hemisphere (Fig. 4*B*). This decrease occurs outside of the Jülich atlas definition of V5/hMT+, close to the superior border of V3 and V4.

**Figure 5.**
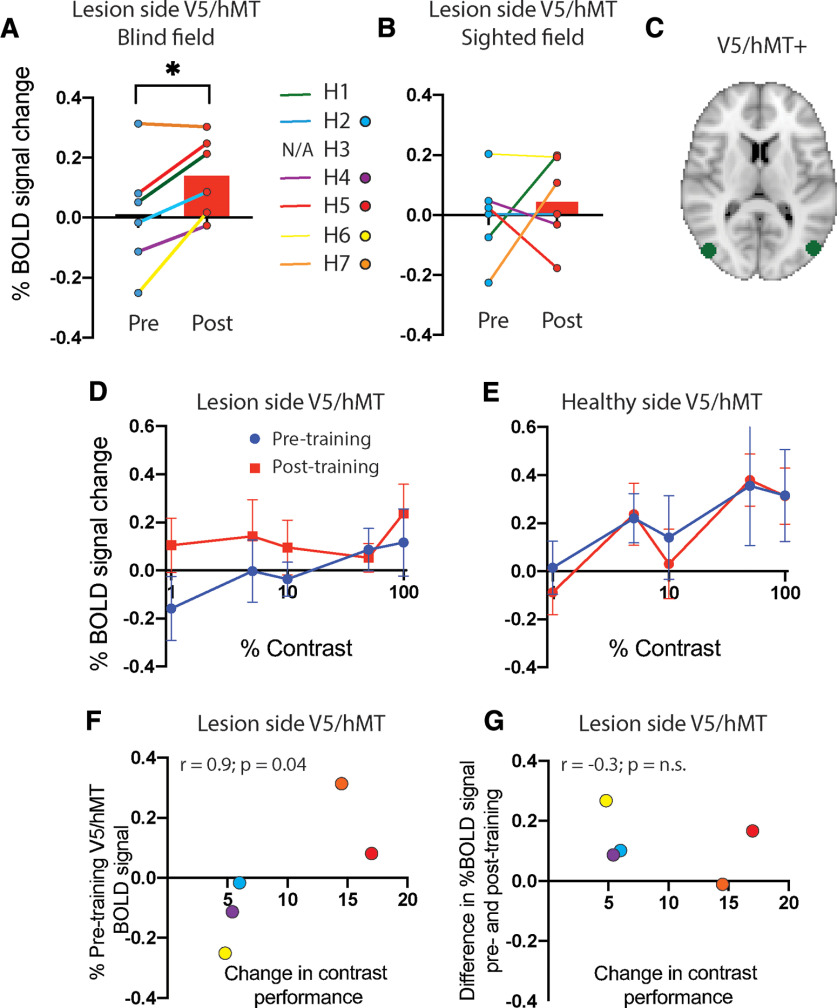
***A***, The percentage of BOLD signal across all contrast values measured in anatomically defined V5/hMT in the lesion side of the brain before (blue) and after (red) training for stimuli in the blind hemifield. Almost all participants showed an increase, showed by the individual paired data points. ***B***, The response in the same visual area to ipsilateral stimulation (presented in the sighted field), which showed no difference before and after training. ***C***, The definition of V5/hMT based on Kolster et al., (2010). ***D***, ***E***, When the BOLD data were divided into the different contrast values, there was a significant effect of training in the lesion side V5/hMT (***D***) but not for the sighted hemifield, measured in the healthy hemisphere (***E***). ***F***, The correlation between the baseline BOLD activity averaged across all contrast levels in lesion side V5/hMT and change in contrast detection performance. Only the five participants not at ceiling pretraining are included, and this small sample shows a significant correlation (Spearman's *r* = 0.90; *p* = 0.04). No such correlation was present between the change in BOLD signal with training and the change in contrast detection performance (***G***).

Reflecting the whole-brain analysis, ROI analysis of V5/hMT showed a significant increase in the BOLD response to the contrast stimulus when averaged across all voxels within the visual mask shown in Figure 5*C* (one-tailed Wilcoxen signed rank test; sum of ranks = 19; *n* pairs = 6; *p* = 0.03). All except one participant showed an increase in BOLD signal post-training compared with pretraining (Fig. 5*A*). In contrast, there was no effect of training in V5/hMT in response to ipsilateral stimulation (Fig. 5*B*).

Considering the change in each contrast individually pretraining and post-training, there was a weakly significant effect of training (*F*_(1,25)_ = 4.2; *p* = 0.05), but no effect of contrast (*F*_(4,25)_ = 0.6) or interaction (*F*_(4,25)_ = 0.6), shown in Figure 5*D*. Figure 5*E* shows data from the intact hemisphere for stimuli shown to the sighted hemifield. The pattern of activity was similar to that of the lesioned hemisphere, albeit with a lower overall level of activity in both sessions.

**Figure 6.**
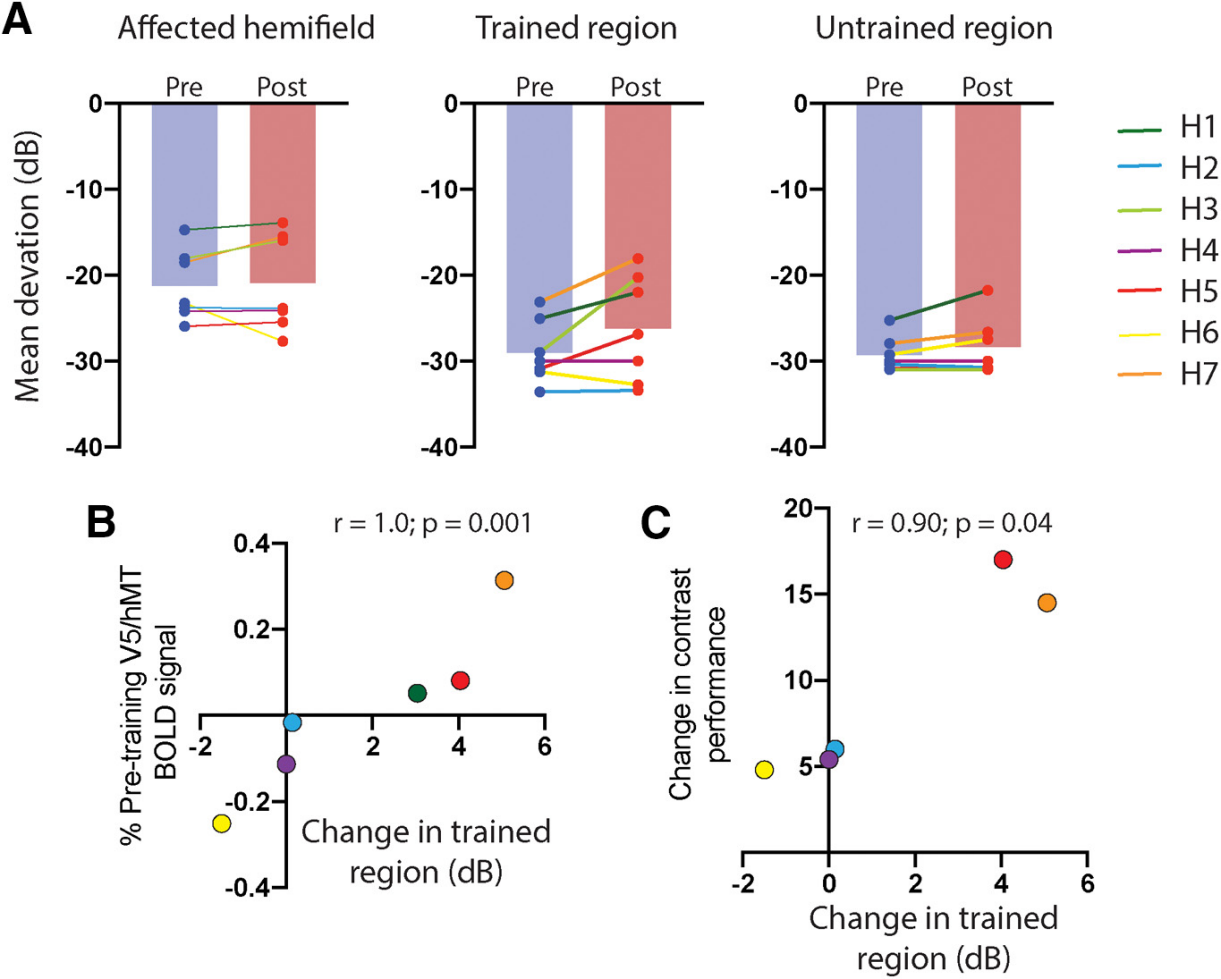
***A***, The Humphrey perimetry mean deviation pretraining and post-training for the entire blind hemifield, the points lying within the trained region, and a control area outside the trained regions. ***B***, The correlation between the baseline percentage of the BOLD signal in V5/hMT and change in visual field within the trained region. ***C***, shows the correlation between the change in contrast detection performance and change in visual field sensitivity within the trained region for the five participants not at ceiling at baseline testing.

In addition to the increase in mean BOLD across all contrasts with training, in the five patients not at ceiling, this signal at baseline (pretraining) correlated significantly with behavioral change in contrast sensitivity (Fig. 5*F*; one-tailed Spearman's *r* = 0.9; *p* = 0.04). V5/hMT signal at baseline also showed a correlation with change in perimetry mean deviation in the targeted region of visual field (Fig. 6*B*; one-tailed Spearman's *r* = 1.0; *p* = 0.001).

### Improvement in static visual fields correlates with change in psychophysical performance in the trained region

Static visual fields in the central 30° were measured before and after training in all participants using Humphrey Visual Field Analyser (program 30–2 full threshold). There was little overall change in either hemifield, indicating a lack of consistent effect across the group. However, it is also the case that the exact training location was not the same for all participants, which is likely to lead to additional variability.

A more detailed analysis (Figure 6*A*) considered the mean change in sensitivity across all points in the affected hemifield before looking specifically at the trained region and an equivalent untrained region in the affected hemifield. There was no difference between the loss in sensitivity before and after training across the hemifield, with some participants improving and others worsening. However, when only points within the trained region were considered, four of the participants showed an increase in sensitivity after training. The remaining three showed little or no change, although across the group there was a significant effect of training (mean gain of 3.4 dB, one-tailed Wilcoxen signed rank test; sum of ranks = 17; *n* pairs = 7; *p* = 0.047). In an equivalent untrained region of the affected hemifield, there was little improvement in any participant.

There was a strong association between training-related changes in visual field sensitivity and contrast detection in the targeted region of vision (one-tailed Spearman's *r* = 0.9; *p* = 0.04), as well as nonsignificant positive correlations with change in speed detection (*r* = 0.53) and speed discrimination (*r* = 0.30). In comparison, there was no association between changes in contrast detection in the targeted region of vision and visual field sensitivity changes in equivalent untrained region of the affected hemifield (*r* = 0.02).

### Improvement in direction discrimination inversely correlates with lesion volume

**Figure 7.**
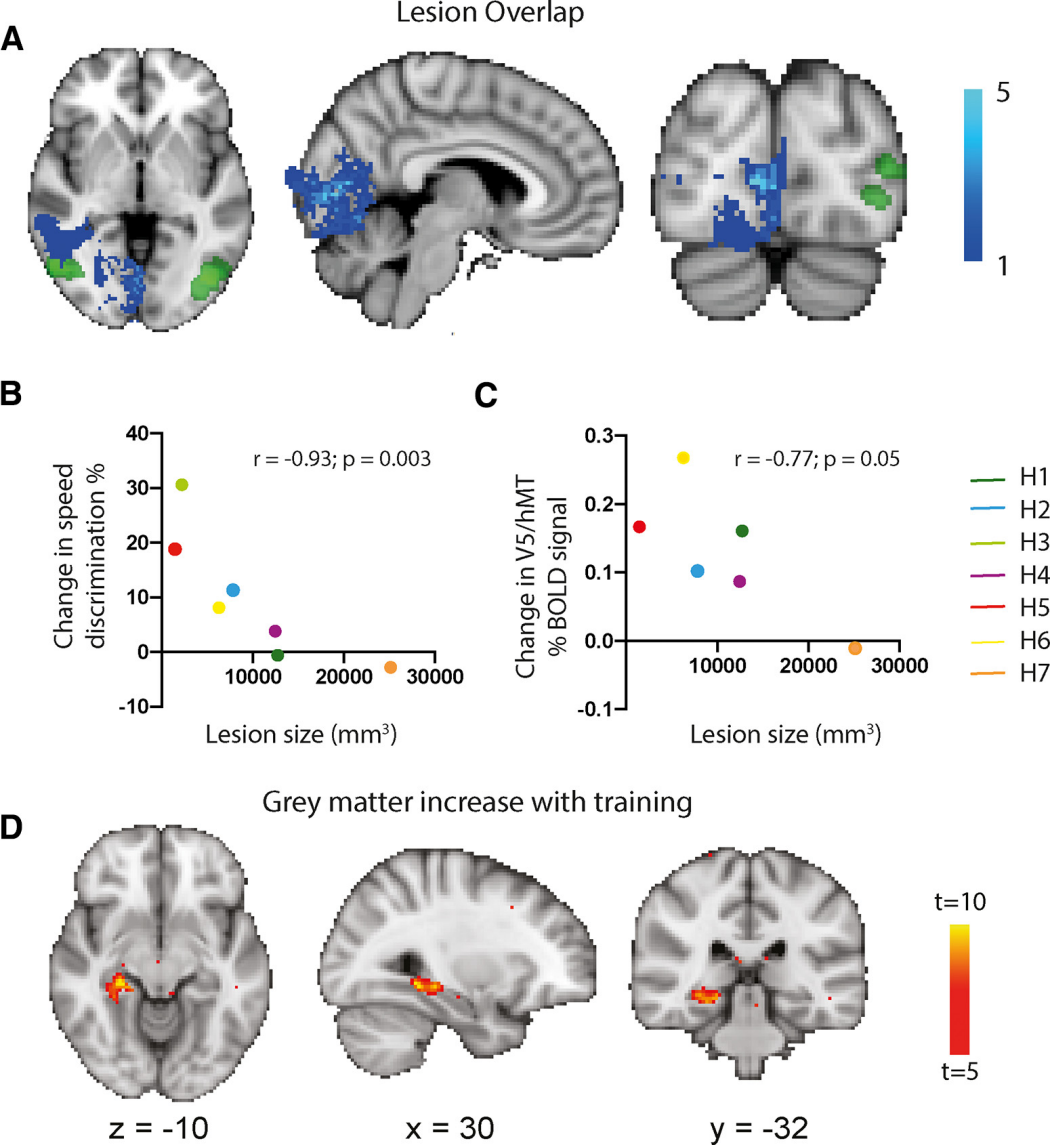
***A*** shows the overlap of the lesions across the seven participants. Only one extends close to the white matter of V5/hMT, with most concentrated around the calcarine sulcus. ***B***, The strong inverse relationship between amount of improvement in the speed discrimination task before and after training and lesion size. ***C***, Relationship between the baseline V5/hMT BOLD signal and lesion size. Each participant is identified by color. ***D***, Increase in gray matter between pretraining and post-training sessions. This was restricted to the hippocampus in the trained hemisphere with no change in gray matter in the occipital cortex on the trained side.

To determine whether there was any link between lesion size and change in visual performance with training, lesion volume was estimated for each participant using previously published techniques (Ajina and Bridge, 2018). Figure 7*A* shows individual lesion masks transformed to a standard MNI template and summed. Lesions were centered around V1, with one participant's lesion extending to the white matter anterior and caudal to V5/hMT. The lighter blue indicates greater overlap, as indicated by the scale bar. Change in performance with training showed a significant inverse correlation with lesion size in the speed discrimination task (one-tailed Spearman's *r* = −0.93; *n* = 7; *p* = 0.003; Fig. 7*B*). The inverse correlation between lesion size and fMRI change in V5/hMT activity pretraining and post-training was also relatively strong (one-tailed Spearman's *r* = −0.77; *n* = 7; *p* = 0.05; Fig. 7*C*). None of the other behavioral experiments, including detection performance and visual field sensitivity, showed a significant association with lesion volume.

### Increase in trained hemisphere hippocampus gray matter volume

To determine whether the visual training increased gray matter volume in the participants, a VBM approach was taken, using a paired *t* test design. Figure 7*D* shows the regions in which gray matter was increased post-training compared with pretraining. The hippocampus on the trained side was the only region showing a significant increase in gray matter. No regions of the occipital lobe on the trained side of the brain showed a change in gray matter volume.

## Discussion

Over the past two decades, significant progress has been made in developing rehabilitation strategies aimed at restoring lost visual functions after brain injury. The evidence to date shows changes in visual performance following systematic and repeated exposure to visual stimuli in active detection and discrimination tasks. This improved performance is demonstrated and tracked using psychophysical methods that are devised to minimize changes in subjective criterion shifts (Sahraie et al., 2006; Huxlin et al., 2009; Das and Huxlin, 2010; Sahraie et al., 2010; Das et al., 2014; Elshout et al., 2016; Melnick et al., 2016). This is important as there has been recent disparity in the region of blind visual field showing improvement on psychophysics deep in the scotoma and the region measured with perimetry, where changes are largely restricted to blind field border zones (Sahraie et al., 2010; 2013; Cavanaugh and Huxlin, 2017; Barbot et al., 2020). Understanding the neuronal changes that mediate improved psychophysical performance is of utmost importance as this can potentially elucidate not only the mechanisms but also the limits and extent of changes achievable with training. For that reason we have conducted an exploratory small-scale study to identify the candidate parameters of interest for future work. Our study shows correspondence between changes in visual sensitivity using psychophysics and perimetry in targeted regions of the blind visual field; albeit perimetry changes are subtle and occur most strongly in patients with greatest baseline extrastriate cortex activity. Together with a correlation with neural imaging activity for stimuli in the same region of the blind field, this suggests an underlying mechanism that is beyond a practice or attentional effect. Even small and focal gains in perimetric mean deviation are considered meaningful in disorders of vision (Chauhan, 2008). The observation that changes in clinical perimetry were specific to the trained region of visual field further suggests that improvement could be more widespread if greater regions of the visual field are targeted.

### Motion area V5/hMT shows an increase in response to Gabor patches following training

The main change in functional activation evident across the group of patients was in V5/hMT, rather than in intact regions of V1. Although the precise retinotopic representation in V1 has been known for over a century, brain lesions because of ischemic (or hemorrhagic) strokes do not often respect physiological boundaries and are likely to extend to neighboring areas as well as the underlying white matter. Any rehabilitation training therefore has to be tailored to the specific deficit and will therefore potentially stimulate neurons in different anatomic locations. Functional imaging studies concentrating on within-subject analysis have shown evidence of changes in V1. Nevertheless, once the changes are combined across individuals at a whole-brain level, any differences in V1 activity will be diluted. Figure 2 shows the considerable variability in lesion location and size across the seven patients in our study, which can explain the lack of measurable functional change in V1.

Because receptive fields are larger and retinotopic maps coarser in extrastriate areas, the precise location has less effect on group analyses. Hence, it is not surprising that some aspects of V5/hMT in the lesion hemisphere show increased activation following training. The change is localized to V5/hMT rather than the larger, more anterior, region that encompasses the human equivalent of MST (Huk et al., 2002). With different training paradigms or different stimulus parameters, even greater changes might occur in this region.

Despite a main effect of increased V5/hMT activity after training, the change in V5/hMT activity did not correlate with change in performance across participants. Blindsight represents a dissociation between motion perception and V5/hMT activity, as neural activity in response to moving stimuli in the blind visual field can be demonstrated in the absence of conscious awareness. Although certain motion responses in V5/MT correlate closely with behavior, such as the perception of globally coherent motion (Newsome et al., 1989)., V5/MT neurons are less modulated by attention or task demands than higher areas such as lateral prefrontal cortex. Therefore it may not be surprising that change in performance does not correlate with a change in V5/MT activity. Instead, we observed a significant correlation between baseline (pretraining) V5/MT activity and improvement in psychophysical performance and clinical perimetry. This suggests that baseline V5/MT function may be necessary for plasticity and may be a useful predictor of who could benefit most from this type of training. Four of our seven participants showed increased sensitivity on perimetry of at least 3 dB in the trained region of the blind hemifield. If this could be predicted by measuring residual function or integrity in the extrastriate cortex, this would permit much more efficient targeting of time-intensive therapies for patients.

### There is some reduction in ipsilateral activity in the lesion hemisphere

An unexpected change in neural activity was that training within the blind field seemed to affect the level of activation in the lesioned hemisphere even when the stimulus is presented to the sighted hemifield. We have previously shown that stimulus presentation to the sighted hemifield leads to activity in V5/hMT+ that is more bilateral than seen in healthy visual systems (Ajina et al., 2015a, 2015b), driven by relatively high ipsilateral activity in the lesioned hemisphere. This activation of the damaged hemisphere during stimulation of the sighted field is indicative of extensive communication between the two hemispheres because of the considerable reduction in input to the lesioned side. A Transcranial Magnetic Stimulation study has previously shown enhanced interaction in blindsight patient GY who could detect moving phosphenes when V5/hMT was stimulated bilaterally, but not when stimulation was restricted to V5/hMT in the damaged side (Silvanto et al., 2007). We observed a reduction in ipsilateral activity post-training that was not in V5/hMT but slightly more posterior at the border of V3/V4. These regions are likely to be interconnected with V5/hMT in the same hemisphere as well as possessing direct callosal connections with the intact hemisphere. Unfortunately, it is not possible to infer how adaptation may have occurred in this instance.

This type of change in hemispheric interactions has been previously described in the motor system following unilateral stroke, and it is not clear whether it is beneficially adaptive or maladaptive (Johansen-Berg et al., 2002). Rehabilitation strategies have attempted to modify this activity and appear to show promising results (Di Lazzaro et al., 2010). It is therefore worth investigating further the role of changing the balance of visual activation in extrastriate cortex in the two hemispheres as a rehabilitative approach. Indeed, a similar finding was evident in a previous neuroimaging study of short-term visual training in hemianopia that used a different population of participants (Larcombe et al., 2018b).

### What is the role of stimulus and training type?

The training stimulus was a vertically oriented Gabor patch, which was similar in size, spatial, and temporal frequency to the stimulus used for behavioral assessments and fMRI acquisition. The only notable difference was the orientation of drift, which was diagonal in behavioral and fMRI protocols. This specificity of test and training stimuli makes it impossible to know whether enhanced V5/hMT activity after training would translate to different stimuli in the blind field. However, the observation of increased detection and discrimination of moving dots post-training, and the correlation between fMRI activity and perimetry makes it likely that the effect of training extends beyond a stimulus-specific effect, consistent with previous studies (Das and Huxlin, 2010; Das et al., 2014).

The changes in visual perception shown here, measured with both psychophysics and visual field measurements, appear to be subtler than seen in some previous studies (Cavanaugh and Huxlin, 2017). The most likely reason for this is differences in the training stimuli as training duration was comparable. The two main ways in which the stimuli differ are (1) the use of Gabor patches rather than moving dots and (2) the requirement to detect the target rather than discriminate between two different stimuli. Because it has previously been shown that improvement in visual function is also evident after training with static Gabor patches, this suggests it is not a limiting factor (Das et al., 2014). This latter training study, however, did require discrimination between horizontal and vertical orientations rather than simply requiring detection. A discrimination task is considerably more challenging to perform in the blind field as it requires not only detection of any stimulus but more targeted information such as direction of motion or orientation. This increased challenge is reflected in the discrimination tasks shown in Figure 3, where performance is considerably lower than in the detection tasks. Detection of motion can remain relatively preserved in the absence of direction selective signals (Pasternak et al., 1985), suggesting that psychophysical performance underlying the two tasks may be supported by different mechanisms and perhaps distinct residual structures. In contrast, discrimination of speed and temporal frequency require sensitivity for motion direction and are likely to depend on a common mechanism (Pasternak, 1987).

Another reason for the more subtle improvement in visual perception in the current study may be lesion size. Five participants regained the ability to detect moving stimuli after training, having initially been at chance (H2, H3, H5, H6, H7). Two participants in particular (H3 and H5) showed consistent improvement in the ability to discriminate motion direction across both experiments. These individuals were also the only participants to perform above chance before training, suggesting something unique to their residual anatomy, perhaps V1 sparing, which could facilitate preserved motion direction discrimination (Petruno et al., 2013; Barbot et al., 2020). Of note, both participants had the smallest lesion volumes at 1472 mm^3^ and 2212 mm^3^ (group mean 9736 mm^3^ ± 8103 mm^3^ SD). Although both lesions encompassed retinotopically targeted V1, there was possible sparing of peripheral V1 as well as the occipital pole (central). The latter corresponds to central fixation and macular sparing in hemianopia. Activity in peripheral nonretinotopically activated regions of V1 is interesting as it has implications for residual vision and rehabilitation of hemianopia. In illusory motion perception, peripheral subregions of V1 are active, although stimulation is outside the neuronal receptive fields (Muckli et al., 2005). This is suggested to occur via feedback from higher visual areas such as V5/hMT, which may be important for perception of both real and apparent motion (Pascual-Leone and Walsh, 2001; Hochstein and Ahissar, 2002; Silvanto et al., 2005a, 2005b). This may account for retained local detail processing supporting direction discrimination in the absence of conscious awareness and has implications for identifying patients most likely to benefit from targeted direction training (Huxlin et al., 2009). It is also the case that when the V1 lesions extend anteriorly toward lateral geniculate nucleus (LGN), they may encroach on direct projections from LGN to extrastriate areas. Such lesions have been shown to limit blindsight performance in both nonhuman primate studies (Schmid et al., 2010) as well as in patients (Sahraie et al., 2013).

### The main structural change is in the hippocampus

Detecting subtle changes in brain structure in individual participants is challenging, given the significant variability in brain shape, cortical thickness, and visual area location among individuals. The main hypothesis, given that V1 was damaged in all participants, was that extrastriate areas would be activated extensively during the long-term training, which has been shown in other learning studies to change gray matter volume measured with MRI (Scholz et al., 2009). However, likely because of the small number of participants and variability of the location of area V5/hMT, there were no significant differences in occipital gray matter associated with training.

Nevertheless, there was an increase in gray matter in the hippocampus, likely because of the learning of the task and improvement in its execution. This is consistent with changes in hippocampus activation following short-term visual perceptual training in healthy participants, although no structural measures were quantified in this study (Larcombe et al., 2018a).

In summary, our investigation in a small cohort of patients has highlighted the complexities involved in attempts to underpin the neuronal substrates of behavioral changes following an extensive restorative approach to visual rehabilitation. Future studies on a larger cohort would also benefit from diffusion-weighted imaging, examining the effect of a lesion on the connectivity of early brain areas. Such data may be useful in predicting the potential response to interventions and the extent of improvements in patients. Although the measurements of visual field are often a good indication of the extent of damage and helpful in therapeutic management of retinal diseases, it is likely that as the potential mechanisms for recovery of vision includes areas with large receptive fields such as V5/hMT, clinical visual fields that are commonly static may not be an appropriate outcome measure to quantify the efficacy of interventions. A functional measure of performance, particularly a measure of patient interaction with the environment, could provide a better outcome measure in future studies. Our findings also suggest that future studies will benefit from measuring intervention-induced changes in the intact hemisphere as well as those in the damaged field.
